# Clinical Lectures on Fever

**Published:** 1855-10

**Authors:** William Stokes

**Affiliations:** Regius Professor of Physic in the University of Dublin


Clinical Lectures on Fever. Delivered in the Meath Hospital, Dublin. By William Stokes, M. D., Regius Professor of Physic in the University of Dublin.

Lecture XIII.—1. Congestion, with more or less consolidation, in cases of what is called "diffuse inflammation," "erysipelatous inflammation," or, by some, phlebitis.

2. Similar or nearly similar conditions (so far, at least, as we are taught by pathological anatomy) in cases of purulent absorption, with or without manifest phlebitis.

3. The intercurrent disease of the lung in cases of the eruptive fevers, when they are of the low, putrid or malignant type.

4. Congestions and semi-consolidations of the lung, as intercurrent affections in typhus fever.

5. Analogous conditions arising in the course of the non-maculated or the so-called "typhoid fevers."

6. The disease occurring in connection with delirium tremens from excess. In such cases, you will often find a group of asthenic local diseases, which are generally seated in the stomach, heart, the bronchial membrane, the parenchyma of the lung, and even the pleura. In some instances we find that the patient has also typhus fever.

7. Rapid, extensive, and complete consoldidation of the lung occurring in the course of a typhus fever. In some instances the patient dies asphyxiated; while, in others, a portion of the pulmonary structures falls into sphacelus, and death takes place with the symptoms of acute gangrene of the lung.

Such are some of the more prominent cases which have been classed under the head of typhoid pneumonia. There is another of which we have had many examples, and yet I do not wish you to take what I am going to say about it in any other way than in the light of suggestions. The case, as I said before, is by no means uncommon. The patient is attacked with the usual symptoms of typhus fever, and he comes into Hospital after two or three days' illness. There is nothing about him to make one think that his disease will not run the usual course of the epidemic of the day, and we are prepared to expect a fever of at least a fortnight's duration. On admission he may have no symptom which would call attention to his chest; but, as early in some cases as the beginning of the fourth, and in others of the fifth day, it is discovered that the upper lobe of one lung is solid, or nearly so. The clavicle is quite dull on percussion, so is the scapular spine, and the dullness extends to the line of the mamma. This discovery has been so often made accidentally, that I am sure many of such cases have passed unnoticed. at least, where the attendant is not well informed as to the insidious nature of typhous local diseases, and does not make it a practice and a duty to examine daily, as far as he can, the condition of every organ.

But the most remarkable circumstance in these cases is, that the constitutional disease seems to be cut short. The expression of fever leaves the countenance; the peculiar color or hue of typhus disappears; the eye becomes bright and intelligent; the tongue cleans; and the pulse comes down to a natural state. And thus we have seen patients so altered in the course of twenty-four hours, that one had some difficulty in recognizing them. All the symptoms of typhus were gone, and nothing remained but the consolidation of the lung. And this, too, is not attended with any notable suffering. There may be a little cough, some dull pain, or an inability to lie on one side; but that is all. The respiration is scarcely, if at all, accelerated; in fact, it would seem that there was no irritation or excitement of the organ; and the case is another proof of how much less the sufferings in disease are connected with the mechanical than with the vital conditions of organs.

This local disease, too, is generally easily managed. A small blister or two, the application of the tincture of iodine, and the exhibition of a little hydriodate of potass, with or without a tonic, will remove it. Indeed, the cure is often so rapid, that I have thought that our remedies had little to do with the result. How are we to look at such cases? That they are not examples of inflammation of the lung is plain; and it appears probable that, if this local disease had not occurred, the patient would have gone through the course of the fever of the day. Does it not seem as if the constitutional disease exhausted itself, as it were, in the production of the local affection, just as, in certain cases of simple variola, we see the fever to subside on the appearance of the pustule? I do not know whether such cases have been observed elsewhere, but of their existence we have had abundant proofs. It is worthy of remark, too, that when we compare these cases with the ordinary forms of typhus, attended with secondary disease of the lung, the local affection is here developed at an unusually early period; and it may be, that, in the more protracted cases of fevers, the nature of which is to develope local affections, the periods of this development and of the cessation of the fever may also be coincident. We do not, however, find that this is so common as to establish a rule. Let us, assuming that these curious cases were really examples of typhus with a secondary deposit, again compare tuem with the more ordinary forms of the disease, and we shall find that they want two important characteristics of the longer fevers; one, the successive production or the simultaneous production of various local diseases; and the other, the occurrence of that secondary inflammation or irritation of the parts in which the deposit takes place. That the latter circumstance is one of great weight in relation to the preventing or delaying of crisis, it is impossible to doubt. As to the case of successive or simultaneous production of local diseases, this, at all events, marks a more severe and complicated disease.

Gentlemen, I will not here enter into the wide subject of crisis in fever; yet, I may point out to you, as a matter well worthy of investigation, the possibility of the occurrence of crisis by other modes than those which are generally enumerated; thus we may have a crisis without sweating, or diuresis, or hemorrhage, or diarrhœa, but which takes place by an internal and silent change in the condition of an organ, and yet a change which will, or may, itself spontaneously disappear.

Here let me warn you against the error which so many fall into with respect to these various cases of disease of the lung arising in the course of some form of constitutional disease or fever. They are usually set down as pneumonia, typhoid pneumonia by some. Now, the name itself would be of little moment if its adoption did not lead to errors in practice. And, although it cannot be affirmed with certainty that in none of these cases is there pneumonia. yet we have good grounds for believing that, in many of them, inflammation, as the term is commonly understood, is either absent from the first, or, if it occurs, that it is only secondary to a special lesion induced by some form of constitutional disease.

It is extremely difficult to present to you any well-defined classification of the various forms of diseases which have been described under the head of typhoid pneumonia, or to draw the line between simple asthenic inflammation of the lungs and those conditions which have been described from an early period under the terms of bilious, putrid, or typhoid pneumonia. And observe that when I make use of the term asthenic pneumonia, we refer more to the condition of the general system than to the activity or inactivity of the local disease. For, so far as local inflammatory action is concerned, there is abundant proof that it may originate and proceed with rapidity, and even with vehemence in the very last periods of life, so that the disease may be sthenic *quoad* the local condition, and yet the case itself be asthenic in reference to the general state of the economy. Much of the confusion with regard to this subject has arisen from the circumstance, that too great weight was attached to the presence of certain physical signs, which were taken as always indicating similar conditions. The succession of the signs of crepitus, dullness, cessation of vesicular breathing, and its replacement by bronchial respiration, has been admitted to indicate a simple pneumonia, in which the local disease is the principal pathological condition, and the fever only a secondary one. But it is certain that this train of phenomena, or some modification of them, may occur under exactly opposite circumstances, the local disease being symptomatic of the fever, and not the fever of the local disease. And there is the strongest reason for believing, that even though the mere anatomical condition of the lung in the two cases be similar, yet there is an essential, a vital difference, and that practically we cannot deal with the local disease in the latter case as if it were an original affection. This applies to all those cases of disease with the physical signs of pneumonia, which are secondary to any forms of fever, whether it be typhus or typhoid, whether it be variola or erysipelas, purulent poisoning of the blood, glanders, malignant scarlatina, or malignant measles. In these cases, even though the physical signs accurately correspond with those of the typical pneumonia, which, by the way, is by no means always the case, we must believe that we are dealing with a special condition of parts, and a condition not only special as compared with ordinary pneumonia, but having also another speciality, namely, that which is derived from the parent malady.

In the present state of our knowledge, gentlemen, we cannot declare that any special anatomico-pathological condition exists by which we can distinguish these secondary diseases one from the other; but we may say this much, that practically they all appear to agree in being indicative of an asthenic state of the system, and that, therefore, the supervention of their physical signs at any period of those various diseases, must not be permitted to divert your attention from the general condition of the patient, or make you proceed to treat a case as one of sthenic pneumonia because it has some, or even all, the physical signs of that condition. Do not suppose that I am taking up your time unnecessarily by insisting on these points, for they lead us directly to deal with one of the greatest, if not the greatest and most wide-spread error in the practice of medicine, namely, the treatment of all local, acute, and febrile diseases as inflammations. Here is a group of acute, local, and febrile diseases, and not only this, but a set of cases exhibiting some or all of the physical phenomena of acute pneumonia; and yet, if we subjected them to the ordinary routine treatment of inflammation, the worst consequences would almost certainly follow. You must learn to look at the antecedents and the accompanying general phenomena of these diseases, and set your face against the adoption of any treatment which is based on the doctrine that they are original inflammations. I believe that the erroneous views to which I allude, are, I am happy to say, every day becoming less and less frequent, (thanks to our improved system of clinical instruction,) and the independent spirit of investigation which now animates so many of our students. Notwithstanding, they are still too often acted upon, and over and over again patients who have enough to contend with as the victims of some fell fever or other constitutional disease are lost, or assisted to their death, by the adoption of a local or general anti-phlogistic treatment, in consequence of the physical signs of a pneumonia being discovered; their stimulants are withheld or withdrawn; tartar emetic, or mercury, or even blood-letting is rashly resorted to; and it often happens that, even though the physical signs of the pneumonia are removed or modified, the patient sinks from the combined effect of the original disease and the exhaustion produced by this ignorant and benighted treatment. I do not think that any of you will fall into these or similar errors after what I have so often said; it will, at least, not be my fault if you do.

Let us now consider what we may term the parenchymatous affections of the lung in fever, or, if you will, the typhus disease of the pulmonary structure. It may be stated, generally, that whatever be the differences in the various cases of this affection in typhus, the local disease follows the general law of other lesions secondary to the fever; that is to say, it agrees with them in its mode of invasion, its latency in the earlier periods of its development, its frequent spontaneous retrocession, and, lastly, in its pathological effects. It is quite true, that, as compared with the best marked examples of acute sthenic pneumonia, it is not wanting in any of the physical signs of that disease taken singly, but it is generally different from it in the order or arrangement, as it were, of these physical signs. And, indeed, I think it is an extremely rare circumstance to observe in the course of a typhus fever the rise, progress, and retrocession of a pneumonia which has passed into hepatization, as we so continually see in ordinary cases of the disease. I have already drawn your attention to those curious cases of consolidation of the upper lobe of the lung; now, whether these be genuine examples of an arrested typhus or not it is difficult to say, but their whole history and progress is very different from that of ordinary pneumonia; and I repeat, that there is nothing more rare than to see in the course of a typhus fever the regular succession of phenomena with which Laennec has made us so familiar, as indicating the several successive stages of an acute pneumonia occurring under what may be called its normal conditions.

The most common case is the occurrence, generally, at an early period, especially in the maculated forms, and often at a later period in the non-maculated or so-called typhoid fevers, of a well-marked crepitating râle in the lower lobes of one or both lungs; it is generally much more extensive and distinctly marked in one lung than in the other. The amount of dullness is seldom very great, and we find the disease, as it were, to linger, and for days together to show no disposition either to produce solidity of the lung on the one hand, or to proceed to resolution on the other. The disease is often quite latent, and is only recognisable by careful physical examination; and its discovery, as you will readily understand from what I have said before, is sometimes an unfortunate circumstance for the patient. In the present state of our knowledge we must believe this condition of the organ to be either the result of a certain amount of typhous deposit into the lung, or of a special state, an inflammation, if you will, which is, however, under the general law of the fever, partaking of its specific character, and capable of spontaneous retrocession. It is seldom attended by pain or hæmoptysis, and it constantly exists without any important modification of the general symptoms of the case.

The second form of the disease is of a more serious character, and seems to be connected either with an original pyogenic disposition, itself secondary to typhus, or we may suppose that the typhoid deposit undergoes a rapid purulent transformation, so that in this way a condition of the lung is rapidly established, having some resemblance to the third stage of pneumonia, as described by Laennec. I have not myself seen a sufficient number of these cases to justify me in speaking very decidedly as to their physical signs, but I think I have seen enough of them to warrant me in believing that the course of the disease is different from that of ordinary suppurative pneumonia. We have not observed the intermediate stage of well-marked hepatization between that which is characterized by the occurrence of early râle on the one hand, and the signs of interstitial suppuration on the other. The complete dullness and the bronchial respiration which accompany the third stage of pneumonia, as described by Laennec; we have not observed, the physical signs being principally a persisting râle passing from a fine into a large crepitus, and semi-dullness on percussion. On dissection, the lung is found soft, friable, of a greyish-red color, but still very permeable to air, though infiltrated with purulent matter. It is as if the purulent secretion took place coincidently with or immediately after the first or congestive stage. Some of the patients have had sweatings and a sanguinolent and somewhat sanious expectoration; but we have not hitherto observed the ordinary prune-juice sputa in these cases. I have seen this disease in connection with purulent deposits in the neck, and posterior mediastinum, but it may occur without the formation of purulent matter in any situation other than the lung; it may supervene in the advanced periods of the case, and at a time when the patient seems about to recover; or it may come on much earlier, and when the skin is thickly covered with the petechial eruption. The last case is the most formidable; though one attended with the greatest danger, the disease is, however, not always fatal, and we have had several cases in which recovery took place. I need not say that they were all treated upon a tonic and stimulating plan, in addition to which we employed dry cupping and blisters.

The last case of which I shall speak to-day is by far the most formidable of the pulmonary affections of typhus; it is characterized by the sudden, complete, and singularly extensive consolidation of the lung. In the course of twenty-four, or even, sometimes, of twelve hours, the most extensive and complete dullness may be produced in a lung which had been previously free from physical signs, or, at most, had only exhibited some of the ordinary bronchial râles of typhus. We have thus the signs of complete hepatization, unpreceded by the crepitating râle; the disease begins by consolidation, and then one of two results follow: either the patient dies speedily, generally with loose râles in the opposite lung, combined with tracheal effusion; or, after a day or two, he begins to expectorate a horribly fetid matter, and we discover by the stethoscope that a large cavity has formed in the lung. This is a true gangrenous cavity formed in the very center of the solidified mass, and the disease has a close pathological analogy to the process of acute mortification which has been described as occurring in some of the worst cases of the typhous disease of the intestinal glands.

Let us now pass in review the circumstances in which these forms of disease occur; for, when we compare them with the ordinary conditions of acute primary pneumonia, we cannot but admit that they indicate a lesion of a very different nature.

In the first place, the physical signs are preceded by fever; and it may not be till many days have elapsed that the symptoms of lung affection, as it were, spontaneously arise. Secondly, the fever is obviously an essential fever; it may occur with or without petechiæ, and other complications may or may not be present. Thirdly, the disease sets in without any apparent external exciting cause. Fourthly, when the purulent form is observed, it appears to be, as it were, the second stage of the affection; and I may here remark, that on dissection, we rarely, if ever, find what is termed concrete or non-concrete purulent matter. Lastly, the invasion of one form of the affection may be sudden, and the signs of extensive and complete consolidation be among its earliest phenomena. It is in this case, too, that if time be allowed, large eschars forming cavities, which may commmunicate with the bronchial tubes, are liable to occur.

I may remark here, that in two cases of this rapid consolidation, the gangrenous eschar did not communicate with the bronchial tubes. One was a case of typhus, in a man who had long before suffered from gangrene of the opposite lung; the other occurred in a case of what is termed the erysipelatous or diffuse inflammation.

---

Lecture XIV.—I endeavored to convey to you, at our last lecture, that the conditions which have been described under the head of typhoid pneumonia were probably examples not only of a pathological but of an anatomical state of parts different from that which is found in the simple original inflammation of the lung. And it is a great deal easier to say what they are not than what they are, to state their negative rather than their positive characters.

Now I wish to state to you here that a certain change has occurred in our opinions as to the origin of the so-called typhoid inflammation of the lung. We at one time held that it was the coexistence of gastritis or enteritis which gave to the pneumonia the typhoid character. This view was held by us before we had, by that imperceptible power of conviction which arises from experience, admitted the two following principles in their entirety:

1. That symptoms which are diagnostic of local disease, where the patient has not an essential fever, are either altogether valueless or much lessened in value when such a condition exists; and

2. That the gastric or gastro-enteric lesion is rare even as a secondary disease in fever; so that when irritation of the structures of the intestinal tubes occurs it is a remote, tertiary, and accidental phenomenon.

Our present opinion on this matter is in general the following: That in cases in which there are, in connection with the signs of typhoid lesion of the lung, evidences of gastro-intestinal disease, both the pulmonary and abdominal lesions spring from the one parent condition, and that so far from the specialities of the pulmonary being derived from the accidental complication with the abdominal disease, both have a common character originating in the same source, I am quite sure that a large proportion of those cases described as asthenic pneumonia depending on gastro-intestinal complication, have been examples of essential fever, with the two affections coexisting as secondary lesions.

We have seen that in these cases, I will not say of typhoid pneumonia, but of typhous or typhoid affections of the lung, the various physical signs of pneumonia, singly considered, may be present, and are actually often to be found. They fail, however, very frequently to present themselves in the regular order or succession which is observed in true acute pneumonia.

Now let us inquire whether there is any physical sign peculiar to these cases of typhous pulmonary affections, which does not occur, at least as the rule, in ordinary pneumonia; and I do not know of the existence of any such, unless it be the sign of tympanitic resonance over the diseased lung, a condition first noticed by Dr. Hudson, of this city, and to which he attaches some importance. Dr. Hudson states that in certain cases of typhoid consolidation of the lung, the sound on percussion was very different from that observable in the ordinary condition of hepatization. He describes it as "a tympanitic clearness over the solidified lung without air being present in the pleura;" indeed he goes so far as to say that in one case the tympanitic clearness on percussion existed fully to the same degree and of the same kind as in pneumo-thorax; here the lung was found perfectly solid throughout, with the exception of a small extent of the anterior and postero inferior parts which was still crepitating.

It is very difficult to understand what condition of parts could have caused this singular tympanitic clearness over a solidified lung. When we speak of tympanitic resonance, it must be always borne in mind that the tympanitic sound does not always imply clearness on percussion. When you have a cavity in the center of a solidified lung, or when you have hepatization of the left lung, in connection with flatulent distention of the stomach, the sound, on percussion, though dull as compared with that of the healthy lung, has a distinctly tympanitic character; to this we have long been in the habit of giving the name of tympanitic dullness. I have never found it, however, to simulate the tympanitic resonance which occurs in pneumo-thorax, or in dilatation of the air cells; it is inferior in degree, and different in character. Dr. Hudson met with four cases, in which the observation of this phenomenon was followed by dissection. One was that of a man who died of extensive inflammation of the left lung in the Meath Hospital, in the spring of 1832, in which, at the close of the case, from the hollow sound on percussion at the lower part of the left side, it had been previously quite dull, a pretty general opinion existed that a pneumonic abscess had formed and burst into the pleura. On dissection, the side having been punctured, no air escaped; the lung was red and solid, but without abscess, and the pleura was adherent over two-thirds of its extent. I must, in justice to myself, state that in this case I never entertained the idea that an abscess had opened into the pleura. In another case the lung was found hard and solid from chronic pneumonia; and in the two remaining cases the condition of parts was similar to that which was presented by that first detailed.

Dr. Hudson admits that in three of those cases the tympanitic sound might be explained by reference to the distended state of the stomach; but in the fourth he thinks that the explanation might be found in the facility with which the vibrations of the air in the bronchus, and its larger divisions, may be supposed to be communicated through a lung in that condition, that is to say, solid throughout; and, therefore, not permitting the loss in a mixed medium of solid and healthy lung of such vibrations.

I am quite prepared to admit that with extensive solidification of the lung, the dull sound, on percussion, may yet have a tympanitic character; but I have seen no case in which this sound could be confounded with that of pneumo-thorax, or of dilatation of the air cells. With reference to the bearings of this question upon the signs of typhoid pneumonia, I can only at this moment remember two cases which are worth detailing to you. In one tympanitic dullness did occur over the diseased portion of the lung, without our being able to account for it, by any accumulation of air, either in the pleura or in the stomach. The case was of a low, putrid character, and I remember suggesting it as a matter just possible that there might have been a typhoid pneumatosis developed in the diseased lung; but I am sure that we were not able to establish the existence of such a condition on dissection: the case occurred a good many years ago.

In the second case, which was one of manifest typhus fever, the posterior portion of the right lung became solid or nearly so, while the anterior face of the organ preserved its vesicular respiration. Now we found that over this portion of the chest, that is, over the front of the thorax on the right side, the sound, as compared with that over the opposite lung, was morbidly clear; it was true tympanitic clearness, not dullness, and it continued for three or four days, and gradually disappeared with the resolution of the posterior solidity: this case occurred in the Hospital last year, and was seen by Dr. Hudson himself. I confess I am quite at a loss to explain the nature or mode of production of this phenomenon. My friend Dr. Lyons mentions to me that in a case of asthenic pneumonia, occurring in a patient of intemperate habits, which we saw in consultation, the anterior superior part of the left lung presented for a couple of days a condition of morbid clearness, but subsequently became engaged in the general consolidation of the organ.

Dr. Lyons is disposed to regard the abnormal clearness which occurs in these cases as the result of the increased pressure of the respiratory column of air in the still permeable portions of the pulmonary cells, which he considers in certain cases become from this cause expanded beyond their natural volume. His views are that the inspired air presses with a certain force on the whole pulmonary surface, and that if a portion of this service becomes impermeable to air from solid deposite, occlusion of the tubes which forced it, or other cause, the remaining portion of the pulmonary tissue is acted on by the whole of the inspiratory force, before which it is thus made to expand; this portion of the lung may thus be considered to be in a condition of temporary emphysema, and so gives a correspondingly clear sound on percussion.

There is a circumstance in connection with the resolution of these typhoid or typhous diseases of the lung, different from what is commonly observed in sthenic pneumonia. You know that the true inflammatory hepatization rarely disappears in a sudden manner. It subsides gradually, and the transition-state between dullness and clearness on percussion is generally marked by the 'crepitus redux.' In the cases before us, however, and especially where the disease is secondary to typhus fever, the resolution, as I have before stated to you, is often singularly rapid, and is often unattended by the crepitus of resolution. If, then, you consider the state of solidification simply, we find it on the one hand to form without the crepitus of the first stage of pneumonia, and on the other to disappear rapidly, and without the rule of resolution. Thus we are permitted, as it were, to witness the silent and spontaneous development and retrocession of one of the secondary diseases of typhus.

This change from the state of consolidation to that of permeability to air, this rapid change, unattended by the crepitus and resolution, probably shows that the real disease was one unconnected with inflammation, either as a primary or a reactive condition.

You will remember that I suggested to you that some of the cases which have been described as typhoid pneumonia, might be held as examples of an aborted typhus. These were characterized by early consolidation, early disappearance of the typhous state, and often spontaneous subsidence of the local disease. I cannot help thinking that between such cases, and those in which the general disease runs its usual course, there is another class or category of cases in which the progress of the merely pulmonary disease is marked, more or less, by signs of irritation or inflammation of the lung, which inflammation or irritation is either reactive or specific, or both reactive and specific. And I apprehend that these cases which, as it were, float between the aborted and the perfect typhus, are much more numorous than might be supposed; and in such instances the case is often treated throughout, without a suspicion of its being really an example of typhous disease having been entertained.

What has been now said should impress on your minds that rule in practice which I have so often urged upon you, namely, that the rules of diagnosis of local inflammatory disease which are good in ordinary cases, lose their value in a great measure when the patient has typhus fever. This was long ago proved by the researches of Louis on the condition of the brain in fever, and it was the non-recognition of this fact which constituted one of the greatest errors in the system of Broussais. I have told you that if you gained nothing during the session but the knowledge and full appreciation of this great principle, your time would have been well spent. How many cases have we not had of headache, delirium, watchfulness, or its opposite, coma—yet without encephalitis; and so it is with the remaining cavities—symptoms of functional alteration are met with in connection with the cerebral, pulmonary, circulating, and digestive systems in fever. They may or may not be attended by organic change, and that organic change, when it does exist, is not necessarily inflammation; and we cannot, I believe, lay down any satisfactory rule of diagnosis which would show that in one case of local functional disturbance there was organic change, and in another that there was not. But this much we do know, that those groupes of symptoms which are diagnostic of local inflammation in a case which is not fever, cease to be so when they occur in a case of typhus. Let this principle be ever present to your minds, for it is impossible to exaggerate its value. Long ago it was acted on empirically by the best physicians, who refused to adopt antiphlogistic measures in treating the local symptoms in typhus, and who employed stimulants irrespective of them, when the general condition seemed to demand such treatment. It now comes before you as the result of an extended and accurate pathological investigation, and the study of the pulmonary phenomena, as we have seen, enables us to go a step further, and to declare that not only are the symptoms of local irritation doubtful or illusive, but that even the physical signs of a pneumonia, when occurring in a case of typhus, are not to be taken as proof that a local inflammation has occurred.

If these things be true so far as our typhus is concerned, it would appear probable that in other acute diseases, under the influence of a law of periodicity, and, perhaps, in many that arise from the operation of an introduced poison, the same circumstances may be found, so that we might apply to a much larger circle of diseases those principles as to the secondary local affections, which appear applicable to typhus fever.—*London Lancet.*

